# Comparison of cardiometabolic risk factors between obese and non-obese patients with nonalcoholic fatty liver disease

**DOI:** 10.1038/s41598-023-41893-w

**Published:** 2023-09-04

**Authors:** Zahra Yari, Danial Fotros, Azita Hekmatdoost

**Affiliations:** 1 Department of Nutrition Research, National Nutrition and Food Technology Research, Institute and Faculty of Nutrition Sciences and Food Technology, Shahid Beheshti University of Medical Sciences, Tehran, Iran; 2 Department of Clinical Nutrition and Dietetics, Faculty of Nutrition Sciences and Food Technology, National Nutrition and Food Technology Research Institute, Shahid Beheshti University of Medical Sciences, Tehran, Iran

**Keywords:** Diseases, Gastroenterology

## Abstract

Nonalcoholic fatty liver disease (NAFLD) is closely associated with cardiometabolic abnormalities. This association could be partly influenced by weight, but not entirely. This study aimed to compare the cardiometabolic risk factors between obese and non-obese NAFLD patients, and explored the relationship between adiposity and severity of fatty liver. This cross-sectional study included 452 patients with Fibroscan-proven NAFLD. Anthropometric measurements, metabolic components and hepatic histological features were evaluated. The risk of metabolic syndrome in each body mass index (BMI) category was analyzed using logistic regression. The prevalence of metabolic syndrome was 10.2%, 27.7%, and 62.1% in normal-weight, overweight and obese participants. Regression analysis showed that the risk of metabolic syndrome in overweight and obese NAFLD patients was 3.74 and 4.85 times higher than in patients with normal weight, respectively. Waist circumference (β = 0.770, P < 0.001) and serum concentration of fasting blood glucose (β = 0.193, P = 0.002) and triglyceride (β = 0.432, P < 0.001) were the determinants of metabolic syndrome occurrence in NAFLD patients. Metabolic abnormalities were similar in obese and non-obese NAFLD patients, although, the increase in BMI was associated with an increased risk of metabolic syndrome in patients.

## Introduction

Non-alcoholic fatty liver disease (NAFLD) is one of the most common causes of hepatic disorders, defined by the presence of steatosis in ≥ 5% of hepatocytes in the absence of excessive alcohol consumption or other known liver diseases^1,2^. NAFLD represents a spectrum of chronic liver diseases, starting with simple steatosis and progressing towards non-alcoholic steatohepatitis (NASH), cirrhosis, and ultimately hepatocellular carcinoma^1^. Recently, it has been found that NAFLD can also increase the risk of extrahepatic cancers, for example, bladder cancer, which has a high prevalence in the elderly population and is associated with NAFLD through insulin resistance^3^. NAFLD, with a global prevalence rate of nearly 25%, has been considered a global public health concern^2^. This prevalence has been reported up to 70% in diabetic and obese patients^2^.

NAFLD, independent of other risk factors, is highly associated with an increased risk of metabolic disorders including cardiovascular disease, type 2 diabetes mellitus (T2DM) and metabolic syndrome (MetS) in obese and non-obese individuals^4,5^. Lean NAFLD has also been shown to be a stronger risk factor for the incidence of T2DM compared to obesity without NAFLD or MetS^4^. As recent meta-analysis has publicized that the risk of MetS and T2DM in non-obese NAFLD patients is 5.43 and 4.81 times higher than non-obese counterparts, respectively^6^. Also, 7-year follow-up of obese and non-obese NAFLD patients revealed that the incidence of cardiometabolic complications is the same in both, which indicates the need for close monitoring^7^. Therefore, cardiometabolic comorbidities seems to be independent of obesity^8,9^. However, although the prevalence of NAFLD is growing substantially in the non-obese population, it has mostly been studied in obese individuals^10,11^.

Although non-obese NAFLD patients have been shown to share clinical outcomes with their obese counterparts^12^, there is not enough evidence on the cardiometabolic status and disease severity in lean NAFLD. Also, the differences in the characteristics of obese and non-obese NAFLD patients remain poorly characterized. This cross-sectional study intends to compare the separate and combined cardiometabolic status in obese and non-obese patients with NAFLD.

## Methods and materials

### Subjects and study design

From 2019 to 2021, 452 Fibroscan-proven NAFLD patients were enrolled in the present study and their clinical data were collected prospectively. The inclusion criteria for this cross-sectional study were as follows: (1) adults 18–65-year-old; (2) Fibroscan findings confirming fatty liver grade ≥ 2, and (3) willingness to participate in the study. In this study, we excluded the subjects with (1) significant alcohol consumption (> 30 g/day); (2) history of treatment for viral hepatitis; (3) diagnosis renal failure, malignancies, infectious disease, chronic liver disease other than NAFLD; and (4) receiving effective drug treatment for NAFLD and/or bariatric surgery over the past 6 months.

This study was conducted in accordance with the ethical guidelines of the Helsinki Declaration. The written informed consent form was signed and dated by all participants. The study protocol has been approved by the Ethical Committees of National Nutrition and Food Technology Research Institute, Shahid Beheshti University of Medical Sciences.

### Clinical and laboratory evaluations

After recording general information about medical history, medications, alcohol consumption, and smoking habits, all patients underwent laboratory testing, physical examination and liver assessment. Height and body weight were measured without shoes and in light clothing by a well-trained nutritionist. BMI was calculated as body weight (kg) divided by height squared (m^2^). Waist circumference was measured in centimeters at the minimum circumference between the lower rib and the iliac crest to the nearest 0.1 cm using an inextensible metric tape. Blood pressure was measured in seated position, after 15 min rest, by using a standard mercury sphygmomanometer.

Venous blood samples were collected after a 12-h overnight fast to measure serum levels of aspartate aminotransferase (AST), alanine aminotransferase (ALT), gamma-glutamyl transpeptidase (GGT), total cholesterol (TC), triglycerides (TG), high density lipoprotein-cholesterol (HDL-C), low density lipoprotein-cholesterol (LDL-C), fasting blood sugar (FBS) and insulin. All laboratory parameters were assessed by commercial kits (Pars Azmoon, Tehran, Iran) using standard methods.

Enrolled individuals were classified based on BMI as normal weight (< 25 kg/m^2^), overweight (25–30 kg/m^2^) or obese (≥ 30 kg/m^2^)^13^. Participants who met at least three abnormalities of the following metabolic syndrome criteria recommended by National Cholesterol Education Program’s Adult Treatment Panel III (NCEP: ATP III)^14^ were categorized as unhealthy metabolic phenotype: (1) abdominal obesity, defined as waist circumference (> 102 cm in men and > 88 cm in women), (2) high systolic/diastolic blood pressure ≥ 130/85 mmHg, and/or the current use of anti-hypertensive medication, (3) FBS ≥ 100 mg/dl and/or current treatment with anti-diabetic medication, (4) low HDL-C concentration (< 40 mg/dl in men and < 50 mg/dl in women), and (5) triglycerides ≥ 150 mg/dl and/or the current use of lipid-lowering drugs.

### Liver assessment

NAFLD was diagnosed through FibroScan® (Echosens, Paris, France) equipped with XL probe, after exclusion of heavy alcohol consumption, viral, or other chronic liver disease. This examination was carried out by experienced hepatologist according to manufacturer’s protocol. Accordingly, fibrosis was measured in kilopascals and scored with a 6-grade scale, from normal to cirrhosis and severe fibrosis. Steatosis was reported in decibels per meter (dB/m) and graded from 0 to 3.

Moreover, the following formulas were applied to estimate the fatty liver in the study:

Lipid Accumulation Product (LAP)^15^: :

$$\begin{aligned} & \left( {Waist\; \, circumference \, \left( {cm} \right) \, {-} \, 65} \right) \times \left( {TG \, \;concentration \, \left( {mmol/l} \right)} \right) \, for \, \;men \\ & \left( {Waist \, \;circumference \, \left( {cm} \right) \, {-} \, 58} \right) \times \left( {TG\; \, concentration \, \left( {mmol/l} \right)} \right) \, for\; \, women \\ \end{aligned}$$

Fatty Liver Index (FLI)^16^:

$$\frac{\left(\mathrm{e}0.953\times \mathrm{loge}\left(\mathrm{triglycerides}\right)+0.139\times \mathrm{BMI}+0.718\times \mathrm{loge}\left(\mathrm{GGT}\right)+0.053\times \mathrm{waistcircumference}-15.745\right)}{\left(1 + \mathrm{e}0.953\times \mathrm{loge}\left(\mathrm{triglycerides}\right)+0.139\times \mathrm{BMI}+0.718\times \mathrm{loge}\left(\mathrm{GGT}\right)+0.053\times \mathrm{waistcircumference}-15.745\right)}\times 100$$

Hepatic Steatosis Index (HSI)^17^:

$$8 \times \left( {ALT/AST\; \, ratio} \right) + BMI \, \left( { + 2, \, if\; \, female; \, + 2, \, if\; \, diabetes \, \;mellitus} \right)$$

Fatty Liver Score (FLS)^18^:

$$1.18 \times \, metabolic \, \;syndrome \, + 0.45 \times \, diabetes \, \left( {2, \, if\; \, yes; \, 0, \, if\; \, no} \right) \, + 0.15 \times \, FSI \, \left( {\text{mU/L}} \right) \, + 0.04 \times \, AST \, \left( {\text{U/L}} \right) \, - 0.94 \times \, \left( {AST/ALT} \right) \, - 2.89$$

BMI, Age, ALT, TG score (BAAT) = was calculated as the sum of the following categorical variables^19^:

$$BMI \, \left( { \ge 28 \, = \, 1, \, < 28 = \, 0} \right), \, age \, at \, liver \, biopsy \, \left( { \ge \, 50 \, years \, = ; \, < \, 50 \, = \, 0} \right), \, ALT \, \left( { \ge \, 2N \, = \, 1, \, < 2N \, = \, 0} \right), \, and \, \;serum\; \, triglycerides \, \left( { \ge \, 1.7 \, \;{\text{mmol/l }} = \, 1, \, < \, 1.7 \, = \, 0} \right) \, thus\; \, ranging\; \, from \, 0 \, to \, 4.$$

(All patients had triglycerides < 400 mg/dl).

### Statistical analysis

All the statistical analyses were carried out using SPSS software, version 20.0 (SPSS Inc., Chicago, IL, USA). Two-sided P value less than 0.05 was considered statistically significant. Smirnov–Kolmogorov test was used to check the normality of our data. Continuous variables are presented as mean ± standard deviation (SD), and compared by one-way analysis of variance (ANOVA). Categorical variables are presented as frequency and percentage, and compared by Chi-square test.

Both univariate and multivariate logistic regression models were applied to calculate odds ratios (ORs) and 95% confidence interval for occurrence of metabolic syndrome in each category of BMI. In multivariate logistic regression models, we adjusted potential confounding factors, including age, sex, steatosis and fibrosis score.

### Ethics approval and consent to participate

The Ethical Committee of Shahid Beheshti University of Medical Sciences approved the study protocol in accordance with the Declaration of Helsinki. All patients signed an informed consent form and the aims and procedures were explained to them.

## Results

### Clinical and biochemical characteristics according to BMI classification

The clinical and biochemical characteristics of participants stratified by BMI are listed in Table 1. Among the 452 NAFLD patients (mean age 45 ± 11.88 years; women 47%), 82 were classified as normal weight, 121 as overweight and 249 as obese. The mean age of patients in different BMI classes was not significantly different. Nearly half of the men (49.8%) and more than half of the women (61%) were obese. There was a stepwise elevation of systolic blood pressure, serum levels of ALT, AST, GGT, FBS and LDL-C with the increase in BMI. Although, there were no significant differences among three BMI classes in terms of biochemical parameters, except for FBS and HDL-C. The frequency of patients with diabetes mellitus (DM) and metabolic syndrome (MetS) increased significantly along with BMI (P < 0.001). Systolic and diastolic blood pressure did not differ significantly between the three BMI classes. Interestingly, patients with a BMI of less than 25 showed the highest severity of fibrosis and steatosis. Along with increasing weight and BMI, the severity of fatty liver also increases. All indices including LAP, FIL, HIS, FLS and BAAT were significantly higher in obese patients than patients with BMI less than 30 (P < 0.001).

**Table 1:** Clinical and biochemical characteristics of participants stratified by BMI.

	BMI < 25 n = 82	BMI = 25–30 n = 121	BMI ≥ 30 n = 249	*P* value
Age, years	44.46 ± 12.25	44.02 ± 10.92	45.65 ± 12.22	0.425
Sex, n, %				0.006
Male	41 (17.2%)	79 (33.1%)	119 (49.8%)	
Female	41 (19.2%)	42 (19.7%)	130 (61%)	
Weight, kg	66.31 ± 10.03	80.91 ± 9.43	98.62 ± 17.85	< 0.001
Height, cm	168.47 ± 11.76	171.13 ± 9.87	164.68 ± 10.04	< 0.001
BMI, kg/m^2^	23.24 ± 1.22	27.56 ± 1.38	36.31 ± 5.58	< 0.001
Waist circumference	90.43 ± 3.97	101.07 ± 6.71	106.39 ± 7.08	< 0.001
Visceral obesity (%)	41.5%	72.7%	92.4%	< 0.001
Systolic blood pressure	134.79 ± 16.52	135.84 ± 19.98	136.74 ± 18.3	0.693
Diastolic blood pressure	84.7 ± 11.37	88.56 ± 13.35	86.55 ± 13.42	0.111
Serum biochemistry tests				
ALT, U/l	32.12 ± 25.31	36.96 ± 32.08	37.45 ± 32.33	0.462
AST, U/l	26.64 ± 24.78	27.84 ± 19.61	29.06 ± 21.94	0.717
GGT, U/l	32.34 ± 28.4	32.04 ± 21.28	36.97 ± 24.99	0.286
FBS, mg/dl	99.11 ± 15.74	104.77 ± 14.52	106.12 ± 19.19	0.007
Triglyceride, mg/dl	160.32 ± 63.43	172.41 ± 68.81	172.39 ± 64.36	0.320
Total cholesterol, mg/dl	188.25 ± 42.8	195.55 ± 36.11	193.94 ± 39.75	0.706
LDL-C, mg/dl	122.81 ± 34.34	131.31 ± 39.28	131.24 ± 42.22	0.227
HDL-C, mg/dl	41.52 ± 9.05	40.12 ± 9.12	37.9 ± 8.26	0.002
DM%	11.4	30.4	58.2	< 0.001
Metabolic syndrome %	10.2	27.7	62.1	< 0.001
Liver histology				
Fibrosis score (kPa)	7.14 ± 2.09	5.92 ± 1.59	6.97 ± 2.71	0.003
Steatosis score (CAP)	326.08 ± 26.48	299.26 ± 33.79	318.23 ± 37.87	< 0.001
Liver indices				
LAP	52.59 ± 23.03	75.11 ± 35.11	87.5 ± 36.24	< 0.001
FLI	34.68 ± 14.19	62.74 ± 16.9	84.07 ± 11.38	< 0.001
HIS	44.75 ± ‌ 25.66	41.92 ± 10.30	50.86 ± 11.98	< 0.001
FLS	− 1.41 ± 1.6	− 0.11 ± 1.11	0.42 ± 1.54	< 0.001
BAAT	1.3 ± 0.88	1.53 ± 0.9	2.25 ± 0.85	< 0.001

Mean ± SD for continuous variables and frequency (number or percentage) for categorical variables.

*BMI* body mass index, *ALT* alanine aminotransferase, *AST* aspartate aminotransferase, *GGT* γ‐glutamyltransferase, *FBS* fasting blood sugar, *LDL‐C* low‐density lipoprotein cholesterol, *HDL‐C* high‐density lipoprotein cholesterol, *DM* diabetes mellitus, *CAP* controlled attenuation parameter, *LAP* Lipid Accumulation Product, *FLI* Fatty Liver Index, *HIS* Hepatic Steatosis Index, *FLS* Fatty Liver Score, *BAAT* BMI, age, ALT, TG score.

### Association between BMI and risk of metabolic syndrome

The association between the risks of metabolic syndrome in each category of BMI was assessed using logistic regression. As indicated in Table 2, crude and full adjusted analysis showed that the risk of developing metabolic syndrome increases with increasing BMI. In Model 4, after adjusting all confounders including age, sex, steatosis and fibrosis score, it was shown that the risk of metabolic syndrome in patients with BMI above 30 and patients with BMI 25–30 is 4.85 times and 3.74 times higher than those with BMI less than 25, respectively (P trend < 0.001).

**Table 2:** Odds and 95% confidence interval for occurrence of metabolic syndrome in each category of BMI.

	BMI < 25	BMI = 25–30	*P* value	BMI ≥ 30	*P* value	*P* trend
Model 1	1 (ref)	5.1 (2.74, 9.49)	< 0.001	8.17 (4.64, 14.37)	< 0.001	< 0.001
Model 2	1 (ref)	5.6 (2.96, 10.71)	< 0.001	8.2 (4.61, 14.62)	< 0.001	< 0.001
Model 3	1 (ref)	3.19 (1.42, 7.19)	0.005	4.69 (2.32, 9.47)	< 0.001	< 0.001
Model4	1 (ref)	3.74 (1.6, 8.74)	0.002	4.85 (2.36, 9.99)	< 0.001	< 0.001

Model 1: Crude.

Model 2: Adjustment for age, sex.

Model 3: Adjustment for steatosis and fibrosis score.

Model 4: Adjustment for age, sex, steatosis and fibrosis score.

Linear regression analysis (Table 3) after adjusting all confounders including age, sex, steatosis and fibrosis score, showed that waist circumference (β = 0.770, *P* < 0.001) and serum concentration of fasting blood glucose (β = 0.193, *P* = 0.002) and triglyceride (β = 0.432, *P* < 0.001) were the determinants of metabolic syndrome occurrence in NAFLD patients.

**Table 3:** Association of metabolic syndrome components and BMI.

	B	SE	β	*P* value^1^
Waist circumference	1.4	0.074	0.770	< 0.001
Fasting blood sugar	0.679	0.213	0.193	0.002
Triglyceride	0.491	0.065	0.432	< 0.001
HDL-C	− 0.114	0.060	− 0.090	0.051
SBP	0.047	0.128	0.018	0.711
DBP	0.044	0.091	0.023	0.630

^1^Adjustment for age, sex, steatosis and fibrosis score.

*HDL‐C* high‐density lipoprotein cholesterol, *SBP* systolic blood pressure, *DBP* diastolic blood pressure.

**Figure 1:**
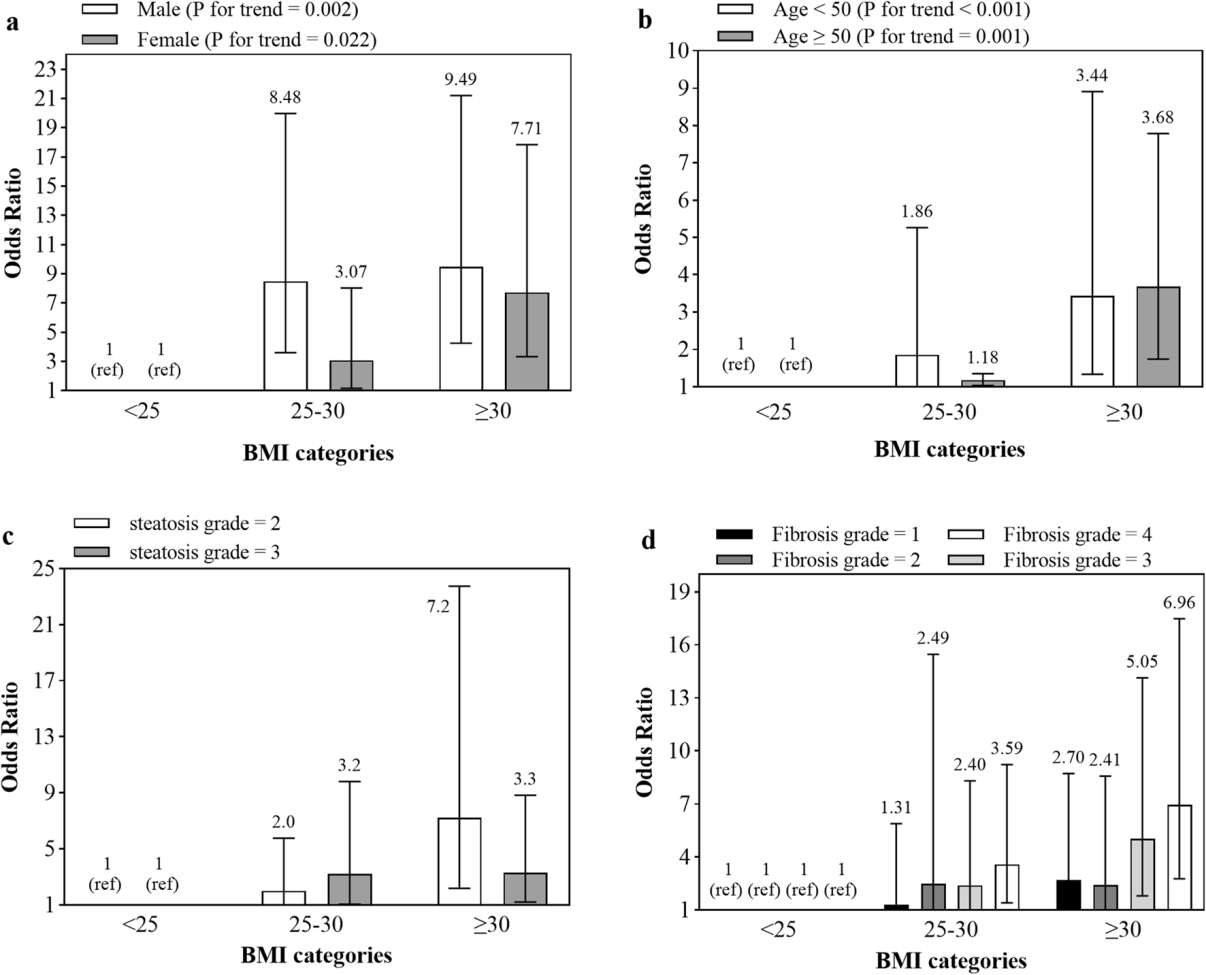
Risk of metabolic syndrome by increasing BMI removed influence carried by sex, age, and steatosis and fibrosis grade.

We calculated risk of metabolic syndrome by increasing BMI removed influence carried by sex, age, and steatosis and fibrosis grade. As shown in Fig. 1, regardless of gender, age, grade of steatosis, and fibrosis, an increase in BMI is significantly associated with an increased risk of metabolic syndrome. However, ORs in male and patients under 50 years old group were slightly more compared with female and patients upon 50 years old group. This risk also increases with increasing grade of steatosis and fibrosis.

## Discussion

This cross-sectional study was performed to elucidate the differences in metabolic features of obese and non-obese NAFLD patients. Our results disclosed that both obese and non-obese NAFLD patients shared several clinical and laboratory characteristics, although MetS was more prevalent among obese participants. In addition, according to the analysis, the risk of metabolic syndrome and the severity of fatty liver increase along with increasing BMI. These results may have significant clinical implications.

Obese patients with metabolic syndrome accounted for the largest percentage (68%) of the present study population. However, NAFLD is also seen in lean people, but it is usually asymptomatic and remains undiagnosed^20^. Previous studies have reported that fasting blood sugar, HbA1C, insulin resistance, and blood pressure are lower in non-obese NAFLD patients^21–23^. Contrary to this evidence, in the present study, no significant difference was observed between obese and non-obese patients in terms of metabolic syndrome components. About half (41.5%) of lean NAFLD patients had abdominal obesity, their mean systolic and diastolic blood pressure was higher than normal and they had dyslipidemia. Interestingly, they also showed higher scores of fibrosis and steatosis.

Although in the general population non-obese NAFLD patients have been shown to share clinical outcomes with their obese counterparts, comparisons of the cardiometabolic risk profile of non-obese NAFLD compared to obese NAFLD have yielded conflicting results^12^. In patients with type 2 diabetes, it was shown that the cardiometabolic risk profile of those with non-obese NAFLD was no better than their obese counterparts. Also, interestingly, cardiometabolic disorders in non-obese women with type 2 diabetes compared to obese female patients showed a stronger relationship with NAFLD^12^. On the other hand, serum concentration of residual lipoprotein cholesterol (RLP-C), an indicator of cardiovascular disease, has been shown to have a worse prognosis in non-obese individuals. This index is independently associated with the incidence of NAFLD^24^.

The evidence about morbidity and mortality in obese and non-obese NAFLD patients remains contradictory. According to a meta-analysis conducted in 2018, obesity was associated with a worse long-term prognosis in obese NAFLD patients^25^. Conversely, the 2020 multiethnic study reported that15-year cumulative all-cause mortality in non-obese NAFLD patients (51.7%) was higher than that of obese NAFLD patients (27.2%) and non-NAFLD subjects (20.7%)^26^. Investigation of NAHANS III data also disclosed that lean NAFLD was independently associated with all-cause and cardiovascular disease mortality^27^. Although evaluation of prognosis and long-term consequences of lean NAFLD requires further studies, current findings highlight the importance of early diagnosis and treatment of NAFLD in lean/non-obese population.

The presence of metabolic abnormalities in non-obese NAFLD patients may be due to insulin resistance, indicating NAFLD-related adverse outcomes in these individuals. In the present study, the severity of liver fibrosis and steatosis was significantly higher in non-obese patients than in obese patients. About half of lean NAFLD patients in the present study had excess visceral fat and suffered from abdominal obesity. Pattern of visceral fat distribution, regardless of BMI, is associated with unfavorable metabolic consequences in individuals^28^. Since visceral fat is associated with insulin resistance and dyslipidemia, in these patients abdominal obesity is more important than total body fat^28,29^. In agreement with these findings, recent Korean cohort showed that a higher ratio of visceral to subcutaneous fat was associated with an increased risk of fibrosis in NAFLD patients, regardless of their BMI^30^. Slightly higher than average total body fat in Asians, compared to other races, has led to a higher incidence of NAFLD-related metabolic disorders^11^. Visceral obesity may be a possible explanation for these observations, which need be clarified by further studies. Visceral fats cause low-grade inflammatory status and metabolic disorders, including metabolic syndrome and NAFLD, by recruiting pro-inflammatory macrophages^31,32^.

One of the strengths of this study was using a Fiberoscan by an expert hepatologist to diagnosis NAFLD and histological examinations. Relatively complete laboratory information allowed a complete comparison of metabolic status between patients. Despite these strengths, the results of this study should be interpreted with caution due to the following limitations: First, due to the cross-sectional nature of the study design, the causality of relationship between fatty liver, metabolic syndrome, and abdominal obesity could not be confirmed. Second, normal-weight patients remain relatively small percentage of the population. Third, it was not possible to assess body composition to accurately determine fat mass. Forth, the inclusion criteria might lead to biased results, because patients with a NAFLD grade1 were not included in the study. Therefore, further large-scale studies with gender, steatosis and fibrosis grade and body composition will be required to better elucidate the pathogenesis and features of NAFLD.

## Conclusion

We performed this cross-sectional study to elucidate the metabolic differences of obese and non-obese NAFLD patients. Based on the results of the present study, it can be concluded non-obese NAFLD patients discloses a similar degree of NAFLD histological severity and metabolic abnormalities compared to their obese counterparts. Insulin resistance and abdominal obesity, regardless of BMI, might play a role in the severity of steatosis and fibrosis in patients.

## Data Availability

All data generated or analyzed during this study are included in this published article.
