# Author Correction: Comparison of cardiometabolic risk factors between obese and non-obese patients with nonalcoholic fatty liver disease

**DOI:** 10.1038/s41598-024-51893-z

**Published:** 2024-01-15

**Authors:** Zahra Yari, Danial Fotros, Azita Hekmatdoost

**Affiliations:** 1 Department of Nutrition Research, National Nutrition and Food Technology Research, Institute and Faculty of Nutrition Sciences and Food Technology, Shahid Beheshti University of Medical Sciences, Tehran, Iran; 2 Department of Clinical Nutrition and Dietetics, Faculty of Nutrition Sciences and Food Technology, National Nutrition and Food Technology Research Institute, Shahid Beheshti University of Medical Sciences, Tehran, Iran

Correction to: *Scientific Reports* 10.1038/s41598-023-41893-w, published online 04 September 2023

The original version of this Article contained an error in Affiliations 1 and 2.

Affiliation 1

“Department of Nutrition Research, National Nutrition and Food Technology Research Institute and Faculty of Nutrition and Food Technology, Shahid Beheshti University of Medical Sciences, Tehran, Iran”

now reads

Affiliation 1

“Department of Nutrition Research, National Nutrition and Food Technology Research, Institute and Faculty of Nutrition Sciences and Food Technology, Shahid Beheshti University of Medical Sciences, Tehran, Iran”

In addition,

Affiliation 2

“Department of Clinical Nutrition and Dietetics, Faculty of Nutrition and Food Technology, National Nutrition and Food Technology Research Institute, Shahid Beheshti University of Medical Science, Tehran, Iran”

now reads

Affiliation 2

“Department of Clinical Nutrition and Dietetics, Faculty of Nutrition Sciences and Food Technology, National Nutrition and Food Technology Research Institute, Shahid Beheshti University of Medical Sciences, Tehran, Iran”

The original Article has been corrected.

